# National and Provincial Prevalence of *Pediculus humanus* capitis among Urban Students in Iran from 2014 to 2018

**Published:** 2019-09-08

**Authors:** Ahmad Ziaoddini, Roya Riahi, Motahar Heidari-Beni, Hasan Ziaoddini, Sara Zamani

**Affiliations:** ^1^School of Medicine, Tehran University of Medical Sciences, Tehran, Iran; ^2^Child Growth and Development Research Center, Research Institute for Primordial Prevention of Non-Communicable Disease, Isfahan University of Medical Sciences, Isfahan, Iran; ^3^Department of Health Psychology, Research Center of Education Ministry Studies, Tehran, Iran; ^4^Health Office, Ministry of Education, Tehran, Iran; ^5^Department of Microbiology and Microbial Biotechnology, Faculty of Life Sciences and Biotechnology, Shahid Beheshti University, Tehran, Iran

**Keywords:** Pediculosis, Prevalence, Students, Iran, Urban

## Abstract

**Background:** Pediculosis capitis is one of the most common contagious diseases in overcrowded places, especially in schools. We aimed to determine the overall and seasonal prevalence of Pediculosis capitis (head lice infestation) among Iranian students.

**Study design:** A repeated cross-sectional study.

**Methods:** The present study was conducted among all students, aged 6-18 yr, from urban areas of 31 provinces of Iran from 2014 to 2018. The student’s hair and scalp were examined by trained school health instructor. The prevalence (95% Confidence Interval) of pediculosis were estimated overall and stratified by gender. Linear and Poisson regression models were used for data analysis.

**Results:** Results showed an upward trend of pediculosis from 1.7% to 3.42% during four years of study (*P* for trend=0.006). Overall, the prevalence of this infestation was significantly higher in girls than boys (*P*-value<0.05). The relative risk of head lice infestation in autumn was 2.42 times higher than spring (RR: 2.42, 95%CI: 1.35 to 4.32).

**Conclusion:** Pediculosis is still a health issue among Iranian students, especially in girls. This infestation had upward trend in recent years and was more common in southern Iran. Increasing the awareness of students regarding symptoms and complications of pediculosis and using accurate diagnosis methods can be effective in reducing the prevalence of pediculosis and its consequences.

## Introduction

Pediculosis or head lice infestation caused by *Pediculus humans capitis* ^1^. It occurs both in developed and developing countries and affects millions of people around the world^2^. Pediculosis is more common among students with low socio-economic status and poor hygiene facilitates ^1,3^. Some factors including sex, age, hair features, the number of susceptible individuals, and the time and frequency of close (hair-to-hair) contact associated with the prevalence of pediculosis ^4,5^. Besides, at pediculosis follows a seasonal pattern ^6,7^ for example in a study among children, aged 6-14 yr; pediculosis was more common in winter ^8^. Pediculosis is found mostly in kindergarten, schools, playgroup, and overcrowded places ^4^.

The previous studies reported the varied prevalence of pediculosis among students. its prevalence in the Middle East and other regional countries such as Pakistan, Iraq and Afghanistan from 4.2% to 78% ^1,9-11^. Hitherto studies in Europe reported a wide range of pediculosis prevalence from 1% to 20% ^4,12^. In addition, findings showed the various range of pediculosis prevalence from 1.6% to 67% in different provinces of Iran^1,13-15^. Therefore, the prevalence of pediculosis depends on cultural behavior, society, and climate conditions ^14^.

Pediculosis can lead to various complications such as itching and scalp lesion, secondary bacterial infection, unspecific generalized dermatitis, anemia, and allergic reactions ^2,16^. Moreover, embarrassment, anxiety, considerable discomfort, and disrupt school performance are the adverse effect of pediculosis^11^. Therefore, the epidemiological studies on the prevalence of pediculosis in different region can play an important role in designing programs to control and prevention of this health problem.

We aimed to determine the overall and seasonal prevalence of pediculosis among Iranian students.

## Methods

This was a school-based nationwide study carried out from 2014 to 2018. Iranian students aged 6-18 yr were screened for head lice infestation, from urban areas of 31 provinces of Iran, on average, 12566186 students are available in each school year. School health instructors trained to do hair examinations in each school examined the hair of children and scalp for lice. The hair and scalp were examined by hand separation of the hair every 1-2 cm. The presence of either live or dead eggs/nits regardless of morphologic features or localization was considered pediculosis capitis. In suspected cases, the hair of child was examined by an expert physician. Examination was repeated in different seasons (autumn, winter, spring) each year. Summer was ignored due to school holiday.

Data are presented as prevalence (95% CI) estimated overall and stratified by gender. The linear trend in overall and stratified by gender was assessed by the regression model. A Poisson regression model was used to assess the relative risk of seasonality in overall.

All procedures performed in studies involving human participants were in accordance with the ethical standards of the institutional and/or national research committee and with the 1964 Helsinki declaration and its later amendments or comparable ethical standards. After complete explanation of the study objectives and protocols, written informed consent was obtained from participants.

All analyses were performed using STATA package version 11.0 (Stata Statistical Software: Release 11. Stata Corp LP. Package, College Station, TX, USA). Statistical significant level was considered as *P*< 0.05.

## Results

**Figure 1:**
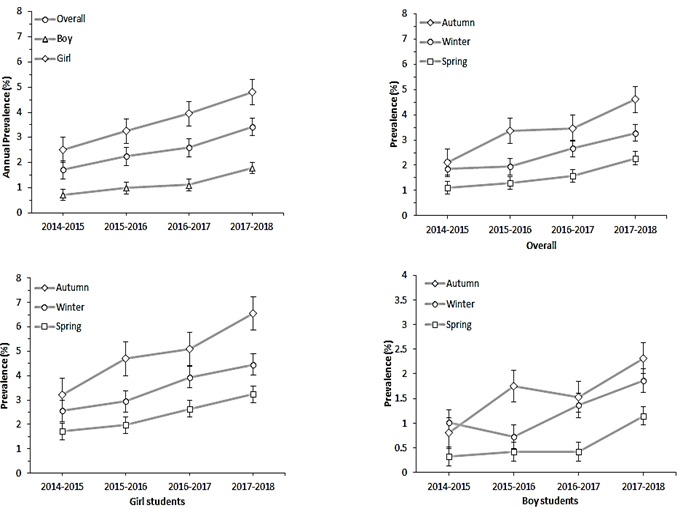
The annual and seasonal prevalence (%) of head lice infestation among Iranian students stratified by gender

The prevalence and trend in the prevalence of head lice infestation among Iranian students according to sex during the four years (2014-2018) are shown in Figure 1. The prevalence of head lice infestation increased significantly over time in both girl and boys (*P* for trend=0.002 for girls and *P* for trend=0.027 for boys). Overall, the prevalence of head lice infestation varied from 1.72% in 2014-2015 to 3.42% in 2017-2018 (*P* for trend=0.006). The prevalence of head lice infestation was higher in girls (2.51% in 2014-2015 to 4.8% in 2017-2018) compared to the boys (0.37% in 2014-2015 to 1.8% in 2017-2018) in each year.

The seasonal prevalence of head lice infestation increased significantly over time in girl students (*P* for trend <0.05), but not in boy students (*P* for trend >0.05). In overall, the prevalence of head lice infestation was higher in the cold season (in autumn: varied from 2.12% to 4.6% and in winter: varied from 1.87% to 3.27%) compare to the spring (varied from 1.10% to 2.28%) in both girl and boy students (Figure 1).

The seasonal relative risk of head lice infestation according to sex is shown in Table 1. The relative risk of head lice infestation in autumn was 2.29 (RR: 2.29, 95%CI: 1.36, 3.87, *P*=0.005) and 3.03 (RR: 3.03, 95%CI: 1.25, 7.38, *P*=0.019) times higher than spring in girl and boys respectively.

**Table 1:** The relative risk ratio of the annual prevalence of head lice infestation among Iranian students for the season according to sex

**Variables**	**Spring**	**Autumn**		**Winter**	
		**RR (95% CI)**	***P*** **value**	**RR (95% CI)**	***P*** **value**
Girls	Ref	2.29 (1.36, 3.87)	0.005	1.51 (0.92, 2.47)	0.090
Boys	Ref	3.03 (1.25, 7.38)	0.019	2.24 (0.93, 5.44)	0.070
Overall	Ref	2.42 (1.35, 4.32)	0.007	1.63 (0.94, 2.85)	0.080

The annual prevalence of head lice according to the provinces of Iran during the follow-up years (2014-2018) are shown in Table 2. The highest prevalence of head lice was observed in Khuzestan Province (7.9%) and the lowest prevalence was observed in Kurdistan Province (0.24%) in 2017-2018.

**Table 2:** Annual prevalence of head lice infestation among Iranian students according to province

**Provinces**	**2014-2015**	**2015-2016**	**2016-2017**	**2017-2018**
Alborz	2.02	1.88	2.69	2.79
Ardabil	0.81	11.34	10.63	6.09
Azerbaijan, East	1.12	1.65	4.26	6.70
Azerbaijan, West	3.29	1.44	9.11	2.75
Bushehr	2.19	2.35	2.87	2.81
Chahar Mahaal and Bakhtiari	0.21	0.35	0.24	0.59
Fars	0.34	0.70	0.85	1.53
Gilan	1.84	2.26	2.93	2.13
Golestan	9.77	5.08	5.61	5.93
Hamadan	0.54	0.23	2.52	2.32
Hormozgān	6.40	4.03	4.13	4.80
Ilam	0.03	0.69	0.55	1.00
Isfahan	0.04	1.88	0.86	0.95
Kerman	2.53	3.04	1.44	1.22
Kermanshah	0.47	0.62	1.31	1.92
Khorasan, North	1.87	2.26	3.06	5.40
Khorasan, Razavi	1.80	3.49	3.99	5.20
Khorasan, South	0.92	0.63	0.70	2.63
Khuzestan	2.10	3.06	5.22	7.90
Kohgiluyeh and Boyer-Ahmad	0.43	7.77	2.70	1.97
Kurdistan	1.45	1.45	0.22	0.24
Lorestan	0.05	1.71	1.52	1.13
Markazi	3.61	1.48	0.84	1.84
Mazandaran	2.37	3.30	1.67	2.81
Qazvin	1.00	1.91	1.94	3.33
Qom	3.51	2.41	2.17	2.36
Semnan	1.65	2.91	2.78	5.15
Sistan and Baluchestan	1.05	6.97	4.29	2.62
Tehran	1.15	2.58	1.74	7.80
Yazd	0.41	0.36	2.32	1.91
Zanjan	0.92	1.92	0.61	0.58

## Discussion

Our nationwide study on Iranian students showed the overall prevalence of head lice infestation varied from 1.7% to 3.4% from 2014 to 2018. This result was approximately similar to previous studies conducted in different regions of Iran, for example, the overall prevalence in Kurdistan province was 4.75% and in East Azerbaijan province was 4.8% ^5,17^, while in some regions of Iran such as southwestern (27.6%) and Qom (13.28%) the overall prevalence was higher than our study ^8,15^. A meta-analysis study on 40 studies among primary school students (aged 5-13 yr) reported that the overall prevalence of pediculosis was 7.4% in Iran^1^.

Head lice infestation affects many people around the world, especially school-age children. The range of worldwide prevalence of this infestation is wide (between 0% to 70%)^18^. Studies carried out in different countries reported distinct prevalence rate ^10,1^. The prevalence of pediculosis capitis during childhood and adolescence in Iran was slightly similar to Korea (4.1%) and was lower than others countries such as Egypt (16.7%, aged 6 to 12 yr), Turkey (27.2% in low socio-economics schools and 13.1% in whole population, aged 5-16 yr), Thailand (23.3%, aged 5-12 yr), and Mexico (13.6% , aged 6 to 16-yr) ^7,10,19-23^. Detection techniques, active screening, season, and geographical condition lead to different results in studies ^7^. On the other hand, socioeconomic and cultural statuses are important factors for these disparities. This infestation is more prevalent in low socioeconomic countries, region with lack of personal hygiene, populous countries ^1,24^.

We found a continuous upward trend for the prevalence of pediculosis during four years of study (from 2014 to 2018) related to the sensitivity of detection methods and precise recording of pediculosis. The influence of diagnosis methods was highlighted by previous systematic examination on school children (6-12 yr) during spring in Germany, true prevalence of pediculosis would have been 3.5 folds higher when wet combing had been used for detection of head lice infestation instead of visual examination ^25^.

Findings of our study showed that pediculosis was more common in Khuzestan province than other proveniences and was less prevalent among Kurdish students. On the other hand, we found that the prevalence of head lice infestation was less common in province with Mediterranean and cold climate such as Zanjan, and Chaharmahal Bakhtiari. These differences between provinces of Iran can be due to different geographical and climate condition, socioeconomic status and personal hygiene status ^11^. Khuzestan with warm and humid weather condition provided a good region for lice aggregation and reproduction. In addition, high population density in a province such as Khuzestan is another risk factor for this infestation ^8^.

Similar to previous studies ^23,26-28^, our findings showed that pediculosis capitis was more frequent among girls compared to the boys (2.67-times more). Study on 8122 Turkish students, aged 5-16 yr, showed that the prevalence of pediculosis capitis in girls was 41-fold more than boys^7^. This difference can be explained by gender-dependence behavioral patterns. Girls prefer to playing with prolonged close contact (head-to-head) and share personal hygiene objects such as combs, hairbrushes, and towels which are the most important ways of passive transmission of pediculosis capitis from one person to another ^6,1^. In addition, having long hair and using hair accessories in girls associated with head lice infestation. short hair in boys provides unsuitable harbor for the reproduction of lice and facilitates detection of pediculosis capitis ^24,27,29,30^.

In the present study, the season had a significant influence on the prevalence of head lice infestation. We found in cold season (especially in autumn), students were more affected by pediculosis capitis than spring. This finding was similar to some previous studies that showed head lice infestation is more frequent in cold weather ^5,8,11,31^. However, a study on students, aged 6-12 yr in Egypt, reported a high prevalence of pediculosis capitis in the warm season (summer) and humid climate^32^. The seasonal variation in pediculosis prevalence can be explained by the influence of climate change on longevity and fecundity of pediculosis capitis and its transmission. For instance, increase in humidity and air temperature can affect the proliferation of lice and facilitates its transmission. On the other hand, in the cold season, high rainfall provides a good condition for the growth of lice. In addition, children wear hot clothes and woolen hat in cold weather that they tend to share these clothes with their sibling and peers or leave them in the schoolroom; these could increase the head lice transmission ^6,8^.

Given that a very large sample of students were considered for this study, we could not have access to more variables such as socioeconomic variables, family size, and long hair which might be the risk factors for head lice infestation.

## Conclusion

The prevalence of head lice infestation was different in seasons and its prevalence in girls was more than boys. The prevalence of pediculosis capitis is still a remarkable pediatrics issue in some provinces of Iran. This infestation was more prevalent in southern Iran and populous provinces. Educating and training the school-age children about symptoms and transmission ways of this infestation is necessary to prevention and treatment of pediculosis. Increasing awareness for precise diagnosis methods can be effective in early detection and reducing the prevalence of pediculosis as well as its consequences.

## Acknowledgements

This nationwide survey was conducted in Iran with the cooperation of the Ministry of Health and Medical Education.

## Conflict of interest

The authors declare that they have no competing interests.

## Funding

None declared.

## Highlights

- Pediculosis capitis is still a health issue among Iranian students, especially in girls.
- The accurate diagnosis method is important in reducing the prevalence of pediculosis.
- The relative risk of pediculosis in autumn was 2.42 times higher than spring.
